# ^1^H-MRS in spinal cord injury: acute and chronic metabolite alterations in rat brain and lumbar spinal cord

**DOI:** 10.1111/j.1460-9568.2010.07562.x

**Published:** 2011-02

**Authors:** Matthias Erschbamer, Johanna Öberg, Eric Westman, Rouslan Sitnikov, Lars Olson, Christian Spenger

**Affiliations:** 1 Department of Neuroscience, Karolinska InstitutetRetzius väg 8, SE-171 77 Stockholm, Sweden; 2 Division of Radiology, Department of Clinical Science, Intervention and Technology, Karolinska InstitutetSE-141 86, Stockholm, Sweden; 3 Department of Neurobiology, Health Sciences and Society, Karolinska InstitutetSE-14186, Stockholm, Sweden; 4 Institute of Neurology, University College London1 Wakefield Street, London WC1N 1PJ, UK

**Keywords:** biochemical profile, experimental spinal cord injury, magnetic resonance, multivariate data analysis

## Abstract

A variety of tests of sensorimotor function are used to characterize outcome after experimental spinal cord injury (SCI). These tests typically do not provide information about chemical and metabolic processes in the injured CNS. Here, we used ^1^H-magnetic resonance spectroscopy (MRS) to monitor long-term and short-term chemical changes in the CNS *in vivo* following SCI. The investigated areas were cortex, thalamus/striatum and the spinal cord distal to injury. In cortex, glutamate (Glu) decreased 1 day after SCI and slowly returned towards normal levels. The combined glutamine (Gln) and Glu signal was similarly decreased in cortex, but increased in the distal spinal cord, suggesting opposite changes of the Glu/Gln metabolites in cortex and distal spinal cord. In lumbar spinal cord, a marked increase of *myo*-inositol was found 3 days, 14 days and 4 months after SCI. Changes in metabolite concentrations in the spinal cord were also found for choline and *N*-acetylaspartate. No significant changes in metabolite concentrations were found in thalamus/striatum. Multivariate data analysis allowed separation between rats with SCI and controls for spectra acquired in cortex and spinal cord, but not in thalamus/striatum. Our findings suggest MRS could become a helpful tool to monitor spatial and temporal alterations of metabolic conditions *in vivo* in the brain and spinal cord after SCI. We provide evidence for dynamic temporal changes at both ends of the neuraxis, cortex cerebri and distal spinal cord, while deep brain areas appear less affected.

## Introduction

Spinal cord injury (SCI) leads to tissue damage with local cell death, demyelination and disruption of axon pathways. Primary injury is followed by an extended period of secondary injury, which may exacerbate the ensuing functional deficits. This process includes inflammation, ischaemia, free radical-induced cell death, glutamate (Glu) excitotoxicity and local tissue remodelling (for reviews, see: secondary injury: Tator & Fehlings, 1991; Park *et al.*, 2004; inflammation: Popovich *et al.*, 1997; Popovich & Longbrake, 2008; free radicals and lipid peroxidation: Demopoulos *et al.*, 1980; complement activation: Hagg & Oudega, 2006; ischaemia: Tator & Koyanagi, 1997; excitotoxicity: Park *et al.*, 2004; astrogliosis/scar: Fawcett & Asher, 1999; Silver & Miller, 2004). Neuroprotective processes and regenerative reactions are also initiated (Hagg & Oudega, 2006; Maier & Schwab, 2006). A degree of spontaneous recovery is typically seen after partial injury to the spinal cord (Narayana *et al.*, 2004). Because cut axons in the adult mammalian CNS typically do not regenerate (Olson, 1997), such recovery is thought to be due to reversal of the spinal shock state and reorganization of existing circuitries both in spinal cord and brain (Frigon & Rossignol, 2006).

Plastic changes after SCI occur in the spinal cord itself, but also in the brain, including cortex and thalamus (Fouad *et al.*, 2001; Hofstetter *et al.*, 2003; Kerschensteiner *et al.*, 2005; Bradbury & McMahon, 2006; Ramu *et al.*, 2007). In the spinal cord, early degenerative changes have been observed within minutes after injury, and regenerative sprouting within 6–24 h. Later, reactive astrogliosis follows, thought to help prevent inflammatory events by containing the damaged zone, protecting spared tissue and helping re-establish the blood–spinal cord barrier (Faulkner *et al.*, 2004). However, this astrocytic scar also contributes to the prevention of axon regeneration (Silver & Miller, 2004).

Magnetic resonance spectroscopy (MRS) is a non-invasive technique allowing metabolic profiles from CNS areas to be obtained *in vivo.*^1^H-MRS has been used to study metabolic changes after SCI in motor cortex (Puri *et al.*, 1998) and thalamus of patients (Pattany *et al.*, 2002) and rats (Likavcanova *et al.*, 2008). ^1^H-MRS of the spinal cord has also been performed in humans (Gomez-Anson *et al.*, 2000; Cooke *et al.*, 2004; Henning *et al.*, 2008) and in rats (Vink *et al.*, 1989a; Zelaya *et al.*, 1996; Silver *et al.*, 2001; Balla & Faber, 2007; Qian *et al.*, 2010). ^31^P MRS of the spinal cord has been used to investigate metabolic changes after SCI in rabbits (Vink *et al.*, 1989a,b;) and pigs (Akino *et al.*, 1997). In this study, metabolic changes were monitored by ^1^H-MRS in acute and chronic SCI, in cortex cerebri, thalamus/striatum and the spinal cord below injury. Group differences were found for specific metabolites. Because our work monitors acute as well as chronic changes simultaneously in distal spinal cord, thalamus/striatum and cortex, we can conclude that SCI leads to metabolic changes at several different levels of the neuraxis, and that there are differences between the acute and chronic condition.

## Materials and methods

### SCI

All experiments were performed according to procedures approved by the Stockholm animal ethics committee. Twenty-eight female adult Sprague–Dawley rats (180–250 g) were operated under isoflurane anaesthesia. Briefly, skin and muscles were opened and a laminectomy was performed at vertebral level T9. After exposure of the spinal cord, a 3-mm-long segment of the spinal cord was removed with a sharp knife, in order to assure complete transection. Muscles and skin were sutured. After surgery, rats were placed on heating pads and received analgesics (buprenorphine, Temgesic, Schering-Plough, Kenilworth, NJ, USA) and antibiotics (Borgal, Hoechst, Warren, NJ, USA) to prevent infections. Assisted bladder emptying was performed twice daily as long as needed. As expected, rats only recovered minimal, presumably reflexive, movements of an occasional joint in the hind limbs, corresponding to a Basso–Beattie–Bresnahan locomotor score below 1 (Basso *et al.*, 1995). Animals were kept from 1 day to 4 months before being killed.

### ^1^H-MRS

Individual rats were scanned using ^1^H-MRS at several time points (Table 1), starting with naïve control rats. Anaesthesia was induced with isoflurane and maintained during scans by spontaneous breathing of 2% isoflurane. Rats were positioned in supine position and fixed to an acrylic rig. Pulse was monitored, and body temperature kept at 37 ± 0.5 ºC with a warm laminar air stream.

**Table 1:** Overview of animals studied at different time points

	Brain			
		
Time after SCI	Bilateral cortex	Thalamus	Unilateral cortex	Spinal cord
Naïve control	Rat 1–10	Rat 1–10	Rat 21–28	Rat 11–20
1 day	Rat 11–20		Rat 21–28	Rat 11–20
3 days	Rat 11–20		Rat 21–28	Rat 11–20
14 days				Rat 11–20
3 months	Rat 1–10	Rat 1–10		
4 months	Rat 1–10	Rat 1–10		Rat 1–10

**Fig. 1:**
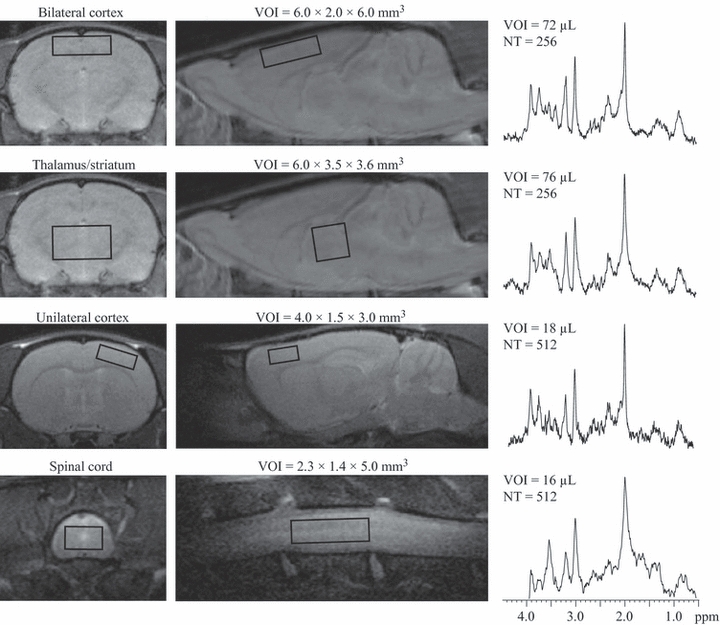
Multislice RARE images of the rat brain with the volumes of interest (VOIs) bilaterally in the cerebral cortex, thalamus/striatum, unilaterally in cerebral cortex and spinal cord, and representative *in vivo* ^1^H-MRS measured from the corresponding brain regions. RARE MRI; in plane resolution: 156 × 156 μm; slice thickness: 1 mm; effective TE: 37.4/25.4 ms for brain/spinal cord; and TR: 2500 ms. Localization sequence PRESS; TE: 16–20 ms; TR: 3500 ms; number of transients (NT): 256–512 and VOI: 16–76 μL.

Table 1 presents an overview of groups of animals, time points and areas from which spectra were acquired. Four different volumes of interest (VOIs) were investigated (Fig. 1), positioned in the cerebral cortex (two VOIs), thalamus/striatum and lumbar spinal cord, respectively. Spectra from all four VOIs were not acquired from the same groups of animals, as stated in Table 1. The cortical analysis was subdivided into a bilateral cortical VOI and a smaller unilateral parietal cortex VOI. The bilateral cortex VOI (72 μL) was placed in the centre of the brain, including motor cortex of both hemispheres, and adjusted so as not to include subdural space, 5 mm behind the rhinal fissure (Fig. 1). This VOI was used to monitor short-term and long-term metabolic changes. The unilateral cortex VOI (18 μL) was centred over the sensorimotor area of the hind limbs (Fig. 1), based on previous results from functional magnetic resonance imaging (MRI; Hofstetter *et al.*, 2003; Endo *et al.*, 2007) to investigate short-term changes. Another VOI (76 μL), including thalamus/striatum and located in the deep centre of the brain, was used to study long-term changes. This VOI was placed 9.0 mm behind the rhinal fissure, positioned between the lateral ventricles (Fig. 1). Spectra were also acquired from a 16-μL VOI in the lumbar spinal cord beneath injury, to monitor both short-term and long-term changes. This VOI was placed five segments below the lesion (Fig. 1). At this level the volume of the rat spinal cord (white and grey matter together), and the proximity of the spinal cord tissue to the body surface allowed reproducible MRS data to be obtained. Using the 13th rib as a landmark, the appropriate level of the spinal cord (Fraidakis *et al.*, 1998) was located. Whenever possible, the VOI was centred between two intervertebral discs, avoiding inclusion of bone lining the vertebral canal and the large vessels on the dorsal and ventral side.

All experiments were performed using a horizontal 4.7 T/40 cm magnet (BioSpec Avance 47/40; Bruker, Ettlingen, Germany) equipped with a 12-cm inner diameter self-shielded gradient system (maximum gradient strength 200 mT/m). A linear birdcage resonator (Bruker, Ettlingen, Germany) with an inner diameter of 35 mm was used for excitation and reception to acquire bilateral cortex and thalamus/striatum spectra). A 72-mm birdcage resonator for transmission and a ‘rat brain’ quadrature receiver coil (Bruker, Ettlingen, Germany) secured to the animal holder above the head was used to acquire the unilateral cortex VOI spectra. For the spinal cord, a surface coil (T9510; Bruker, Ettlingen, Germany) with an inner diameter of 20 mm was used for transmission and detection. *B*_0_ was optimized at the VOI using linear shims.

Voxel shape and localization was achieved by point-resolved spectroscopy (PRESS) using Hermite radiofrequency pulses with matched bandwidths of 5.4 kHz. The voxel displacement due to chemical shift to the peaks discussed in this work can then be estimated to be 8%. The PRESS sequence was applied with the following parameters: repetition time (TR): 3500 ms; echo time (TE): 16 ms for the unilateral cortex VOI and 20 ms for the other VOIs; spectral width: 2 kHz; and number of acquired complex points: 2048. Reliable water suppression was achieved by VAPOR (Tkac *et al.*, 1999) due mainly to its immunity to *B*_1_ inhomogeneities. Thalamus/striatum and bilateral cortex spectra were obtained with number of transients (NT): 256 (15 min); unilateral cortex and spinal cord using NT: 512 (30 min). Positions of the VOIs were based on multislice RARE images in axial, sagittal and coronal planes with the following parameters: effective TE: 37.4/25.4 ms for brain/spinal cord; TR: 2500 ms; RARE factor: 8/4 for brain/spinal cord; spatial resolution: 156 × 156 μm^2^; slice thickness: 1 mm; and number of averages: 1/2 for brain/spinal cord, respectively.

### Quantification

The software package LCModel (Provencher, 1993, 2001) was used for metabolite quantification (Supporting Information Fig. S1). The quantification algorithm of LCModel applies a non-linear regression to fit a basis set to the experimental data. One of the intentions with this study was to simultaneously monitor metabolic changes over time in the brain and in the spinal cord. Oedema in the spinal cord after SCI biased the quantification relative to water, and therefore metabolite concentrations from all VOIs were quantified relative to total creatine [tCR; creatine (Cr) + phosphocreatine (PCr)]. The following 18 metabolites were included in the basis set: alanine, aspartate, Cr, -CrCH_2_ (correction term), γ-aminobutyric acid (GABA), glucose, Glu, glutamine (Gln), glycerophosphocholine (GPC), guanidoacetate (Gua), phosphocholine (PCh), *myo-*inositol (Ins), lactate, *N*-acetylaspartate (NAA), *N*-acetylaspartylglutamate (NAAG), PCr, *scyllo*-inositol, taurine, macromolecules and lipids. Spectral data associated with Cramér–Rao lower bounds (CRLB) ≥ 20% were excluded from further analysis.

### Statistical analysis

The SPSS software package (SPSS, Chicago, IL, USA) was used for statistical analysis. Data for each metabolite were tested for normal distribution (Kolmogorov–Smirnov test) and for homogeneity of variances. The data were then analysed VOI-wise by multivariate analysis of variance (manova; Pillai's trace) followed by separate univariate analysis of variance (anova) on the metabolites. In order to find out which groups (time points) differed, *post hoc* tests (Tukey) were applied. Data were also investigated with multivariate data analysis (MVDA) using appropriate software (SIMCA; Umetrics AB, Umeå, Sweden). Two MVDA methods were applied: principal components analysis (PCA) and partial least squares projections to latent structures – discriminant analysis (PLS-DA). Before PCA and PLS-DA, the data were mean-centred and scaled to unit variance.

PCA is a multivariate projection method designed to extract systematic variation in data. It is useful to recognize patterns including outliers, trends and groups. PCA transforms an original set of correlated variables to a new set of uncorrelated variables, called principal components. The first principal component (t[1]) best approximates the data in a least squares sense and represents the maximum variance direction in the data. A second principal component (t[2]) is computed in a direction that is orthogonal to t[1] and reflects the second largest source of variation. The components are obtained in order of decreasing importance, the aim being to reduce the dimensionality of the data. Two principal components form a plane and when the observations are projected on to this plane a two-dimensional representation (score plot) of the original high-dimensional data is formed. The coordinate values of the observations on this plane are referred to as scores. Corresponding principal component loadings (p[1] and p[2]) can be visualized in order to interpret the pattern in a score plot. The loadings express the orientation of the model plane in the variable space.

PLS-DA is a regression extension of PCA. A Y-matrix is formed that encodes class membership by a set of ‘dummy’ variables (e.g. group one = 0 and group two = 1). PLS-DA then relates the X-matrix, containing the observed data, and Y to each other by a linear multivariate model. The aim of PLS-DA is to create a predictive model that, using linear combinations of the metabolites, best separate groups within the data. Here, leave-one-out cross-validation was applied, to estimate the overall predictive power of the model. The prediction parameter, *Q*^2^, provides an estimate of the predictive power of a principal component. For the models built in this study *Q*^2^ needs to be larger than 0.05. The variable influence on projection (VIP) parameters reflect the importance of terms in the model both with respect to Y, i.e. its correlation to all the responses, and with respect to X (the projection). Terms with large VIP, larger than 1, are the most relevant for explaining Y.

## Results

**Fig. 2:**
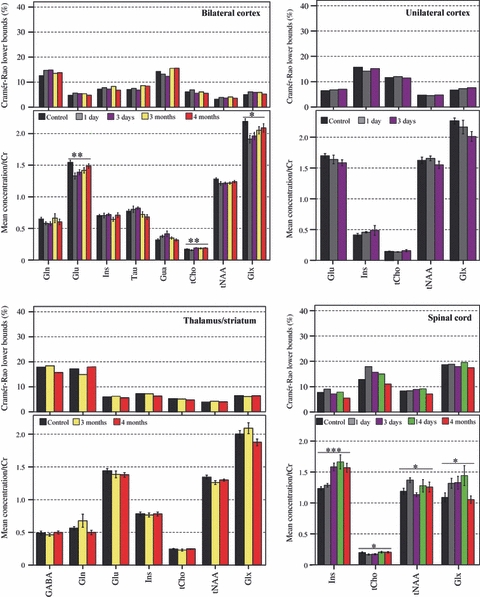
Mean concentrations of brain metabolites and corresponding Cramér–Rao lower bounds (CRLB) quantified by LCModel in bilateral cortex, unilateral cortex, thalamus/striatum and spinal cord. Concentrations quantified relative to total creatine (tCr; error bars = SEM) are shown together with corresponding CRLB expressed in relative units (%). Asterisks indicate metabolites for which significant differences were found over time with anova. GABA, γ-aminobutyric acid; Gln, glutamine; Glu, glutamate; Glx, glutamate + glutamine; Gua, guanidoacetate; Ins, *myo*-inositol; Tau, taurine; tCho, total choline; tNAA, total *N*-acetylaspartate.

Typical locations and sizes of the VOIs centred in bilateral cortex (mean SNR 14.2), unilateral cortex (mean SNR 8.8), thalamus/striatum (mean SNR 12.7) and spinal cord (mean SNR 4.1) are shown in Fig. 1. Corresponding *in vivo*^1^H*-*MRS from the brain regions shown in Fig. 1 represent the spectral quality that was consistently achieved. Shimming resulted in unsuppressed water signal line widths with a full-width at half-maximum of 8–16 Hz. When *in vivo*^1^H-MRS were analysed using LCModel, concentrations of 4–8 metabolites were reliably quantified according to CRLB in cortex, thalamus/striatum and spinal cord of control rats and rats with SCI (Fig. 2). Ins, total choline (tCho), NAA + NAAG (total; tNAA) and Glu + Gln (Glx) were discernable in spectra from all regions studied. In addition, Glu was detectable with low CRLBs in all brain spectra, and Gln in bilateral cortex and thalamus/striatum. Moreover, GABA was detected in thalamus/striatum and taurine and Gua in bilateral cortex. The statistical distributions of data for each metabolite in all studied regions and at all time points were assessed to be normal (Kolmogorov–Smirnov test). manova revealed significant changes over time for the investigated metabolites for the bilateral cortex VOI (*P*= 0.040) and for the spinal cord VOI (*P*= 0.000), but not for the unilateral cortex VOI or the VOI in thalamus/striatum. Figure 2 presents the results of metabolite quantification from the four VOIs that were used to study metabolic changes over time. The mean CRLBs corresponding to the quantified metabolites are also shown in Fig. 2. Asterisks indicate metabolites for which separate univariate anovas showed significant changes over time. Significant differences over time in the bilateral cortex VOI were found, using anova, for Glu (*F*_4,44_ = 3.9, *P*= 0.008), tCho (*F*_4,44_ = 4.3, *P*= 0.010) and Glx (*F*_4,43_ = 3.5, *P*= 0.050). No significant changes were found for any of the detected metabolites in the smaller unilateral VOI in cortex or in the VOI covering thalamus/striatum. In the spinal cord, anova resulted in significant differences between groups for Ins (*F*_4,41_ = 9.7, *P*= 0.000), tCho (*F*_4,40_ = 2.6, *P*= 0.049), tNAA (*F*_4,41_ = 2.6, *P*= 0.050) and Glx (*F*_4,41_ = 2.9, *P*= 0.036).

**Fig. 3:**
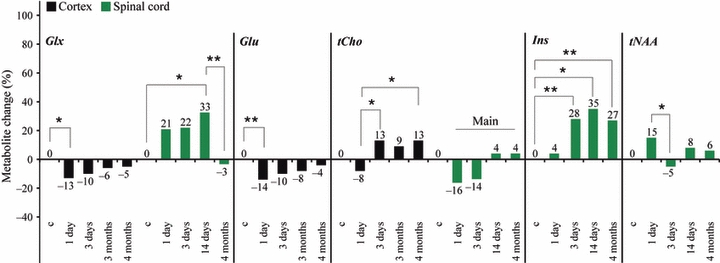
Metabolite changes over time calculated in percent of normal (control rats) for the metabolites that showed significant differences over time (using anova) in this study. Asterisks indicate time points between which *post hoc* tests revealed significant differences. Glu, glutamate; Glx, glutamate + glutamine; Ins, *myo*-inositol; tNAA, total *N*-acetylaspartate.

Figure 3 presents metabolite changes over time calculated in percent of normal (naïve control rats) for the metabolites that showed significant differences between groups. Asterisks indicate metabolites for which *post hoc* tests showed significant differences between time points. A significant decrease of Glx (*P*= 0.013) and Glu (*P*= 0.007) was found between controls and rats 1 day after SCI in the bilateral cortex VOI. Glx in the spinal cord was also found to be significantly different in animals 14 days after SCI compared with both controls (*P*= 0.017) and rats 4 months after SCI (*P*= 0.004). tCho was changed in both cortex and spinal cord. Significant differences were found between animals 1 day after SCI compared with both 3 days after SCI (*P*= 0.011) and 4 months after SCI (*P*= 0.012) in the bilateral cortex VOI. tCho showed significant (*P*= 0.049) differences between groups in the spinal cord with main anova, but not with the *post hoc* tests. Ins was significantly increased in the spinal cord when comparing control animals with animals 3 days (*P*= 0.002), 14 days (*P*= 0.046) and 4 months (*P*= 0.008) after SCI. tNAA showed a significant (*P*= 0.033) difference between animals 1 day after SCI compared with 3 days after SCI in the spinal cord.

**Fig. 4:**
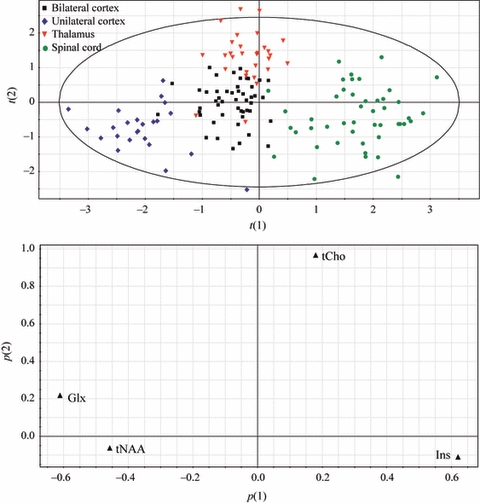
PCA score plots and corresponding loading plots of the two-first principal components in all the VOIs in this study. Each plot mark corresponds to an observation (individual) in the score plots and to a variable (metabolite) in the loading plots. The confidence ellipse is based on Hotelling *T*^2^, at significance level 0.05. Observations situated outside the ellipse are considered to be outliers. t[1] describes differences between spectra from brain and spinal cord. t[2] shows a separation between unilateral and bilateral spectra. The scatter plot reveals that differences caused by the use of diverse coils and VOIs overshadowed differences between controls and rats with SCI. Glx, glutamate + glutamine; Ins, *myo*-inositol; tCho, total choline; tNAA, total *N*-acetylaspartate.

In order to further explore the data set, PCA was performed as shown in Fig. 4. Plots were obtained by drawing the scores and loadings of the first two principal components. The score plot is a summary of the observations (individuals) and the loading plot is a similar summary of the variables (metabolites). The confidence ellipse is based on Hotelling *T*^2^, at significance level 0.05. Observations situated outside the ellipse are considered to be outliers. Each plot mark in the score plots corresponds to one individual and all the metabolite values that were detected for this individual. Plot marks close to each other have similar properties. In Fig. 4 the PCA score plot and corresponding loading plot include data from control animals and animals with SCI from the four investigated VOIs and all time points. Only metabolites that were reliably detected for all the four VOIs as shown in Fig. 2 were included. PCA gave a model, in which the first two principal components explained 75% of the variation in the data. The more important component is t[1], which separates brain spectra from spectra in the spinal cord. A PCA score plot and a PCA loading plot are complementary and superimposable. The loading plot shows which metabolites are influential and how the metabolites are correlated. The further away from the plot origin a variable is, the stronger impact that variable has on the model. Variables contributing to similar information are grouped together; they are correlated. In Fig. 4, Glx and tNAA are grouped together and seem to have similar properties. They are, together with Ins, important for the separation between data from the brain and the spinal cord. The t[2] component demonstrates a difference between thalamus/striatum and cortex spectra. The loading plot demonstrates that tCho is important for this separation.

It was not successful to build robust PLS-DA models for classifications in which data from different VOIs were combined. The PCA score plot in Fig. 4 might provide an explanation of why. The data separate into four clusters that can be interpreted as differences between the four selected CNS areas, but also between the three brain areas on the one hand and spinal cord data on the other, between bilateral and unilateral VOIs, and/or between the different coils that were used in this study. These distinctions overshadowed the differences between classes of animals at different time points. PLS-DA models for classification were therefore built VOI-wise.

**Fig. 5:**
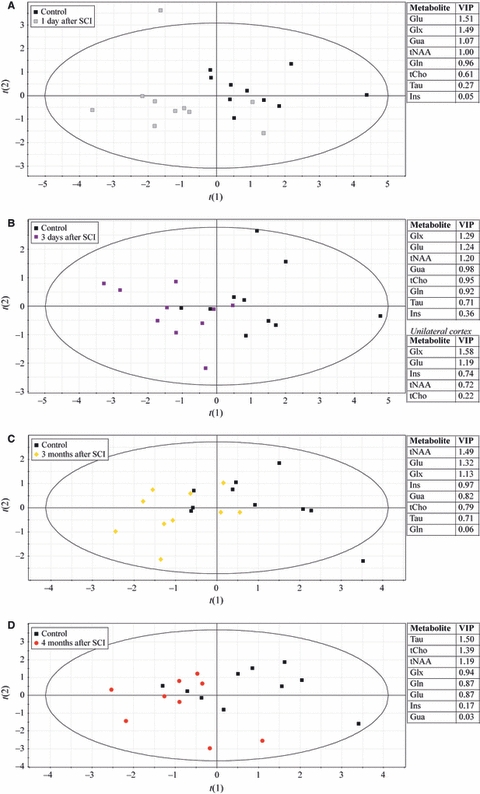
PLS-DA score plots, and corresponding variable influence on projection (VIP) values, of data from the bilateral cortex VOI comparing control rats with rats: (A) 1 day after spinal cord injury (SCI); (B) 3 days after SCI (with VIP values also shown for the unilateral cortex VOI); (C) 3 months after SCI; and (D) 4 months after SCI. t[1] is significant for all models. Gln, glutamine; Glu, glutamate; Glx, glutamate + glutamine; Gua, guanidoacetate; Ins, *myo*-inositol; Tau, taurine; tCho, total choline; tNAA, total *N*-acetylaspartate.

Using PLS-DA, no significant separation between groups was found for thalamus/striatum VOI. Figure 5 shows PLS-DA score plots of cortex data comparing control rats with rats 1 day after SCI, 3 days after SCI, as well as 3 and 4 months after SCI. The model described in Fig. 5A (one significant component and *Q*^2^ = 0.29) resulted in 17/20 correctly predicted rats. Three variables were found to have a VIP value above 1: Glu, Glx and Gua. The model described in Fig. 5B (one significant component and *Q*^2^=0.31) resulted in 16/20 correctly predicted rats. Variables with VIP above 1 were Glx, Glu and tNAA. A model built on data from the unilateral VOI comparing control animals and animals 3 days after SCI also resulted in a significant component (*Q*^2^= 0.26 and 12/16 correct classifications). The score plot of this model is not shown, but the corresponding VIPs are presented below the VIPs of the bilateral VOI. Variables with the two top VIPs are the same as for the bilateral cortex VOI: Glx and Glu. The model described in Fig. 5C (one significant component and *Q*^2^ = 0.26) resulted in 14/20 correctly predicted rats. Variables with VIP values above 1 were tNAA, Glu and Glx. The model in Fig. 5D (one significant component and *Q*^2^ = 0.07) resulted in 15/19 correctly predicted rats. Variables with VIP above 1 were taurine, tCho and tNAA.

**Fig. 6:**
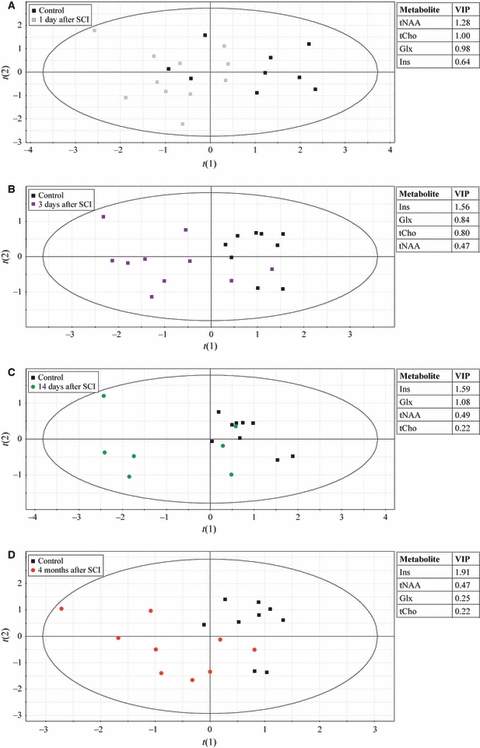
PLS-DA score plots, and corresponding variable influence on projection (VIP) values, of spinal cord data comparing control rats with rats: (A) 1 day after spinal cord injury (SCI); (B) 3 days after SCI; (C) 14 days after SCI; and (D) 4 months after SCI. t[1] is significant for all models. Glx, glutamate + glutamine; Ins, *myo*-inositol; tCho, total choline; tNAA, total *N*-acetylaspartate.

Figure 6 shows PLS-DA score plots of spinal cord data comparing control rats with rats 1, 3 and 14 days, and 4 months after SCI. The model described in Fig. 6A (one significant component and *Q*^2^ = 0.35) resulted in 14/20 correctly predicted rats. The variable with VIP value above 1 was tNAA. The model described in Fig. 6B (one significant component and *Q*^2^ = 0.47) resulted in 15/19 correctly predicted rats. The variable with VIP value above 1 was Ins. The model described in Fig. 6C (one significant component and *Q*^2^ = 0.32) resulted in 13/16 correctly predicted rats. The variables with VIP values above 1 were Ins and Glx. The model described in Fig. 6D (one significant component and *Q*^2^ = 0.38) resulted in 16/18 correctly predicted rats. The variable with VIP value above 1 was Ins.

## Discussion

The present study is a detailed evaluation of the possibilities and limitations of ^1^H-MRS as a tool to monitor CNS consequences of SCI in rodents. MVDA allowed separation between rats with SCI and controls for spectra acquired in cortex and in spinal cord, demonstrating validity of our approach. We compare acute and chronic consequences for the brain and lumbar spinal cord of low thoracic SCI in rats, and find that Glx decreases acutely in cortex and slowly returns to normal levels. Conversely, this metabolite sum increases in the lumbar spinal cord, in which Glx levels remain increased 14 days post-injury, but are normalized 4 months after injury. In cortex, we were able to determine that the Glx decrease was caused mainly by a decrease in Glu. Additional changes of ^1^H-MRS-identifiable metabolites included alterations of tCho in the brain and spinal cord, and of Ins and tNAA in the spinal cord. Of these, the most conspicuous was a marked increase of Ins in the spinal cord below injury, seen first after 3 days and remaining at 4 months, the longest postoperative interval studied. Given the roles of Ins in intracellular signalling pathways, these observations point to marked and long-lasting alterations of cell signalling in spinal cord segments that no longer have bilateral axonal connections with the rest of the spinal cord and the brain. We found that thalamic and striatal tissue appeared less affected by the SCI, because no significant changes were identified in a VOI including these brain areas.

^1^H-MRS has been used to evaluate CNS diseases, including neoplasia, multiple sclerosis, Alzheimer's disease, Parkinson's disease and amyotrophic lateral sclerosis (Lin *et al.*, 2005). For Parkinson's disease it was shown that MRS can assist in both diagnosis and medication of patients (Mueller *et al.*, 2006). Certain metabolites identifiable by MRS have been linked to specific cell populations. Thus, NAA is regarded as solely synthesized in neurons (Moffett *et al.*, 2007), and a decrease is often interpreted as neuronal damage, as found in multiple sclerosis and Alzheimer's disease (Lin *et al.*, 2005). In many diseases, however, it has not yet been possible to detect a specific change of a certain metabolite, but rather a complex time-dependent modulation of several compounds. This led to the concept ‘metabonomics’, defined as ‘the quantitative measurement of the dynamic multiparametric metabolic response of living systems to pathophysiological stimuli or genetic modification’ (Nicholson *et al.*, 1999).

Qian *et al.* (2010) used ^1^H-MRS to study rats with traumatically injured spinal cord. Significant changes in NAA, tCr and tCho levels in segments caudal to the injury were observed. In a study of metabolic changes in thalamus/striatum of rats with SCI (Likavcanova *et al.*, 2008), MRS revealed an increase in the levels of NAA, Cr, Ins and Glu. In sham-operated animals an increase in NAA and Cr levels was found. Decreased PCr values after SCI have been reported up to 4 h in rabbits (Vink *et al.*, 1989a) and up to 6 h in pigs (Akino *et al.*, 1997).

The Glu/Gln/GABA cycle plays an important role in controlling levels of the major excitatory neurotransmitter Glu and the major inhibitory neurotransmitter GABA in the CNS. Gln produced in astrocytes is taken up by neurons, and converted to Glu or GABA. Glu released into the synaptic space is recycled by astrocytes. Excitotoxicity is thought to play a role in the secondary degenerative events that follow SCI (Faden & Simon, 1988; Wrathall *et al.*, 1994; Agrawal & Fehlings, 1997; Park *et al.*, 2004), and increased levels of Glu have been found after SCI at levels neurotoxic even for neurons in the uninjured spinal cord (Liu *et al.*, 1999). To the extent that the marked alterations of Glx in the lumbar spinal cord may reflect alterations of Glu, as seems to be the case in cortex, the Glx increase in the spinal cord could help explain a state of hyperexcitability/increased reflexes often noted after SCI. In cortex, the loss of Glu presumably reflects decreased afferent activity, due to loss of input from hind limbs and other areas below the level of injury.

Ins has a key role in intracellular signalling and has also been regarded as an osmolite and glial marker (Brand *et al.*, 1993; Schuhmann *et al.*, 2003), and increases have been interpreted as increase of glial content or glial proliferation. After SCI, substantial astrogliosis occurs, and starting within 3 days after injury we found a significant increase of Ins in the spinal cord below injury, which was maintained throughout the studied postoperative time. This increase fits with the known long-term increase of glial fibrillary acidic protein-immunoreactivity in most parts of the spinal cord after injury. For group comparison of spinal cord spectra, Ins was clearly the most important metabolite at 3 days and at all later time points. In the brain, Ins levels were fairly stable, and did not change significantly over time. This is in line with the lack of any marked astroglial responses in the brain to SCI.

GPC and PCh are the main components of the prominent choline peak, and constitute metabolites of phospholipid components of cellular membranes. The size of the choline peak is correlated to numbers and densities of cells, increased membrane synthesis or increased membrane breakdown, as in demyelination or glial proliferation (Garnett *et al.*, 2000; Schuhmann *et al.*, 2003). Compared with the changes of Ins, we found only moderate changes of tCho. We noted a 16% decrease of tCho in the spinal cord already 1 day after injury, a time when there was no change of Ins. This suggests that cell membrane changes occur faster in the spinal cord below injury than hitherto appreciated, and that astroglial changes occur as a consequence of the massive axonal damage.

NAA is seen as a prominent peak in the MRS, making NAA one of the most reliable markers for brain MRS studies. Under normal conditions, NAA is synthesized in and exported from the mitochondria, predominantly in neurons, and hence considered a neuronal marker for many brain diseases (Moffett *et al.*, 1993, 2007; Tsai & Coyle, 1995). NAA increases are seen during development (Tkac *et al.*, 2003), presumably due to neuronal maturation and dendritic sprouting (van der Knaap *et al.*, 1990). NAA decreases have been shown by MRS in multiple sclerosis, Alzheimer's disease and Parkinson's disease (Lin *et al.*, 2005; Belli *et al.*, 2006; Yeo *et al.*, 2006; Moffett *et al.*, 2007). An increase of brain NAA has been found in patients suffering from Canavan's disease, a condition in which an enzyme that breaks down NAA is lacking (Bartalini *et al.*, 1992; Moffett *et al.*, 2007), thus providing validation of MRS to monitor brain NAA levels. In a study of patients with incomplete SCI, NAA elevations were detected in the cerebral cortex (Puri *et al.*, 1998). Chromatography-mass spectrometry has also been used to study NAA concentrations up to 1 week after SCI in rats (Falconer *et al.*, 1996). Caudal to injury, NAA levels were virtually indistinguishable from those in control animals. However, despite the wide use of NAA in MRS, its role in the CNS is not fully understood (Moffett *et al.*, 2007). We found an increase of NAA in the lumbar enlargement of the spinal cord following SCI. The increase in the spinal cord was temporary, and might reflect a compensatory upregulation, as NAA is considered to be an important osmolytic regulator for neurons, and/or a regulator of local sprouting. In humans with incomplete SCI (Puri *et al.*, 1998), 50% higher NAA levels were reported 0.5–2 years after injury. The NAA increase at 1 day in the distal spinal cord in our study may represent a protective reaction in the form of increased mitochondrial activity in descending axon arborizations destined to die, leading to a decrease of NAA at 3 days when nerve terminal degeneration is ongoing. At later time points (14 days and 4 months) levels were again somewhat above control levels, possibly the result of increased activity of local neuronal circuits below injury.

Limitations of the technique as performed in the rat include the rather inhomogeneous VOI content in the small rodent CNS. Also, we had to scan five segments distal to the spinal cord lesion, in order for the surface coil to be close enough to cord tissue. The current inability to predict final outcome in spinal injury soon after injury limits how soon after injury novel neuroprotective treatments can be initiated in patients. Future experiments should help in developing increasingly better MRS-based prediction for SCI, especially for the early phase, when it is difficult to predict outcome by other means.

## Conclusion

We show that MRS can monitor metabolic changes after SCI, even in small structures such as the rat spinal cord, and that the method detects changes distant to the site of primary damage, such as in the cerebral cortex with good predictability. As the technology develops, MRS should become helpful in determining the extent of spinal cord damage and its consequences for the brain, possibly assisting in differential diagnosis between spinal shock and permanent injury, as well as for future evaluations of experimental protection and repair protocols for SCI.

## Supporting Information

Fig. S1. Representative LCModel outputs of spectra acquired in bilateral cortex, thalamus. striatum, unilateral cortex and spinal cord.

Please note: As a service to our authors and readers, this journal provides supporting information supplied by the authors. Such materials are peer-reviewed and may be re-organized for online delivery, but are not copy-edited or typeset by Wiley-Blackwell. Technical support issues arising from supporting information (other than missing files) should be addressed to the authors.

## Abbreviations

Cr: creatine
CRLB: Cramér–Rao lower bounds
GABA: γ-aminobutyric acid
Gln: glutamine
Glu: glutamate
Glx: glutamate + glutamine
GPC: glycerophosphocholine
Gua: guanidoacetate
Ins: *myo-*inositol
MRI: magnetic resonance imaging
MRS: magnetic resonance spectroscopy
MVDA: multivariate data analysis
NAA: *N*-acetylaspartate
NAAG: *N*-acetylaspartylglutamate
NT: number of transients
PCA: principal components analysis
PCh: phosphocholine
PCr: phosphocreatine
PLS-DA: partial least squares projections to latent structures – discriminant analysis
PRESS: point-resolved spectroscopy
SCI: spinal cord injury
tCho: total choline
tCr: total creatine
TE: echo time
tNAA: total *N*-acetylaspartate
TR: repetition time
VIP: variable influence on projection
VOI: volumes of interest
